# A minimal ING1b fragment that improves the efficacy of HDAC-based cancer cell killing

**DOI:** 10.1038/cddis.2015.376

**Published:** 2015-12-31

**Authors:** A Boyko, K Riabowol

**Affiliations:** 1 Departments of Biochemistry and Molecular Biology, University of Calgary, Calgary, Alberta, Canada; 2 Department of Oncology, University of Calgary, Calgary, Alberta, Canada

ING1b is a type II tumor suppressor that is inactive in a broad range of cancers, many of which show resistance to apoptosis. It is a stoichiometric member of histone deacetylase (HDAC) complexes^1^ and targets them to the H3K4Me3 histone mark^2^ to regulate transcription. ING1b's effects on transcription are then thought to affect cellular functions relevant to tumorigenesis, including proliferation, senescence and apoptosis.^3^ Many independent studies have reported strong pro-apoptotic effects triggered by ectopic expression of ING1b in cancer cells,^4, 5, 6^ and some studies have suggested that ING1b requires the activity of p53 to effectively induce apoptosis. In a recent study published in *Cell Death Discovery* by Boyko *et al.,*^7^ the minimal polypeptide of ING1b required to efficiently induce apoptosis was defined using a series of deletion mutations followed by different tests to determine their ability to induce cell death. The deletion mutant that showed the greatest apoptotic activity was also tested to determine whether it was able to act synergistically with HDAC inhibitors that target the epigenome, to help define the regions of ING1b that can selectively enhance cancer-cell sensitivity towards cytotoxic agents.

Testing of ING1b fragments was done initially using an early marker of apoptosis, Annexin V staining, combined with flow cytometry for quantitation. Fragments lacking the plant homeodomain (PHD) of ING1b that are responsible for targeting HDAC complexes to H3K4Me3 were as efficient as full-length INGb in inducing membrane flipping as measured by Annexin V staining. This indicated that targeting HDAC complexes and the subsequent effects on transcription were not needed for ING1b-induced apoptosis. However, ING1b fragments containing both an amino-terminal helix and the ING1b nuclear localization sequence that contains nucleolar targeting signals (the NLS/NTS domain) were able to efficiently induce apoptosis at levels similar to the full-length ING1b protein. The helical region includes a previously identified lamin interaction domain (LID), which plays a critical role in maintaining ING1b levels and biological functions in the nucleus by specifically interacting with lamin A.^8^ The NLS/NTS region is required for the subcellular targeting of ING1b to the nucleus and nucleolus^9^ and was also reported to mediate protein–protein interactions required for UV-induced apoptosis.^10^ The study by Boyko *et al.*^7^ confirmed that the localization of ING1b to the nucleus and nucleolus requires a complete NLS/NTS domain and showed that the ability of ING1b to efficiently kill cancer cells requires the amino-terminal helix that is conserved among ING proteins,^11^ as well as a complete NLS/NTS domain containing two NTS motifs.^7, 9^ Therefore, it appears that localization to the nucleus and nucleolus is important for the ING1b peptide, referred to as ING1mini, to induce apoptosis when it is overexpressed.

The cell-killing efficacy of ING1mini was tested using several cancer cell lines (MDA-MB-468, H1299, U-2 OS, Saos-2 and U-87 MG) representing different tumor types. Adenovirus-mediated expression of ING1mini, when used at a relatively low multiplicity of infection (MOI) range, displayed an even higher cell-killing efficiency than native full-length Ad-ING1b. Further analyses using a doxycycline-regulated inducible p53 expression system also showed that apoptosis induced by ING1mini was p53-independent.^7^ Perhaps the most important finding of this study was the ability of ING1mini to act synergistically with HDAC inhibitors in killing cancer cells. A series of combination experiments with Ad-ING1mini and three different HDAC inhibitors, vorinostat (SAHA), panobinostat (LBH589) and trichostatin A (TSA), showed that treatment with any of these HDAC inhibitors increased the cell-killing efficacy of ING1mini significantly. Results for combining ING1mini with LBH589 are shown in Figure 1. The degree of synergism between ING1mini and the drug used was quantified using normalized isobolograms and combination index (CI) calculations. The results of using ING1mini in combination with LBH589 are shown in the graphic, where it is clear that, when combined, cell killing is much more efficient, and indeed is synergistic compared with the effects of either agent alone.

The study by Boyko *et al.*^7^ emphasizes the potential of combined biological and chemical therapies for tumor treatment and indicates that using ING1mini as one component may provide an important therapeutic advantage compared to monotherapy. There is a growing body of experimental evidence that suggests that combining targeted therapies with cytotoxic compounds, including those that induce apoptosis, can improve efficacy, lower the dose of the cytotoxic compound needed and subsequently reduce side effects in patients. In this study, the authors have made the first steps toward developing an ING1b-based therapeutic and provided a rationale for using ING1b-derived therapeutics in combination with existing drug-based epigenetic therapies.

## Figures and Tables

**Figure 1:**
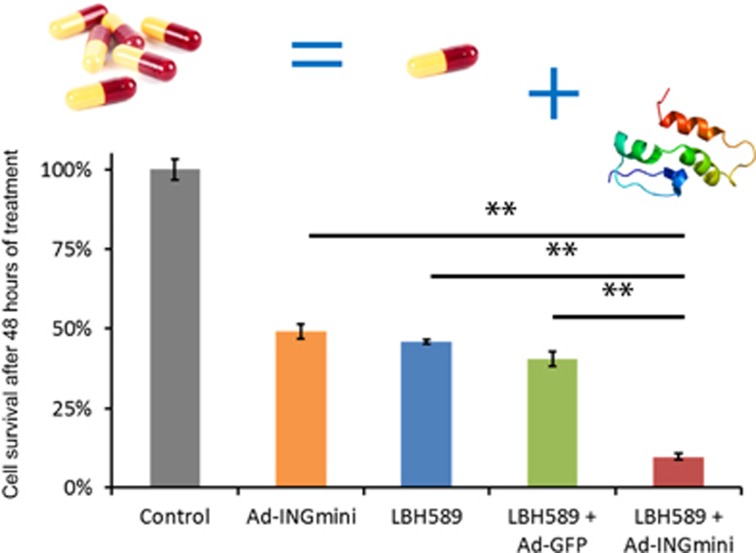
Combining Ad-ING1mini with the HDAC inhibitor LBH589 promotes cancer cell death. MDA-MB-468 breast cancer cells were treated with 100 nM LBH589 alone, or in combination with either Ad-GFP or Ad-ING1mini virus at a multiplicity of infection of 5. Cells grown without virus or HDAC inhibitors served as a control. For combination treatments, cells were first exposed to the drug, and 24 h later infected with the virus and incubated for an additional 48 h in growth medium supplemented with fresh HDAC inhibitor. Cell survival was assessed using an MTT assay. Values represent mean±S.D., *n*=3. **Indicates a significant difference between two means (Student's *t*-test, *P*<0.01).
